# Supplementary material for: Food Waste of Italian Families: Proportion in Quantity and Monetary Value of Food Purchases
Source: Foods. 2021 Aug 19;10(8):1920. doi: 10.3390/foods10081920 (PMC8392151; doi:10.3390/foods10081920)
Supplement: Supplementary file 1 [file foods-10-01920-s001.zip › foods-1318101-supplementary.pdf]

**Supplementary Materials**

**(S1) - Questionnaire S1:**
**The food waste questionnaires developed by van Geffen et al. 2017 (23)**

Thank you for participating in this research.
This questionnaire is part of consumer survey for the European project REFRESH. In this research we would like to ask you a few questions about how food is handled in your household. There are no correct or incorrect answers, it is about understanding your situation. This questionnaire will take about 6 minutes.
In case you fit the target group of this research, you can be in invited for the second part of this research after completing this questionnaire. For this, you will soon be asked to pay attention for one week to what kind of food you have in your household. After this week, we would like to ask you a few more questions.

Are you willing to participate in both parts?
1. Yes
2. No

What is your gender?
1. Man
2. Woman

What is your age?
[answer recorded in years]

What is your highest level of education (completed)?
[Answer options adjusted for the national situation]

In which province do you live?
[Answer options adjusted for the national situation]

Of how many people does your household consist, including yourself?
[Answer in number]

How often do you do the groceries in your household? [screening question]
1. Less than half the times. [screen out]
2. Half the times.
3. More than half the times

How often do you do the cooking in your household? [screening question]
1. Less than half the times. [screen out]
2. Half the times.
3. More than half the times

First of all, we would like to know what you think is important. Regarding food in my household it is important to me that… [items in randomized order, 7-point answering scale ranging from not at all important to extremely important]
1. the food is healthy.
2. the food is tasty.
3. the food is easy to prepare.
4. I have enough food at home.
5. the food is cheap.
6. I don't have too much food at home.

We would like to ask you a few questions about yourself.

What is the size of the place where you live?
[Answer options adjusted for the national situation]

What is your net monthly household income? For this, add up your net income with the net income of your other family members.
[Answer options adjusted for the national situation]

How old are the people you live with? [not reported upon in the current document]
[Multiple answers in years]

How many people usually join the main meal, including yourself? [not reported upon in the current document]
1. ... persons
2. This varies greatly

For each day, please indicate how many persons usually join the main meal: [not reported upon in the current document]
1. Monday: ... persons
2. Tuesday: ... persons
3. Wednesday: ... persons
4. Thursday: ... persons
5. Friday: ... persons
6. Saturday: ... persons
7. Sunday: ... persons

What are the XXX numbers of your ZIP-code?
[Answer options adjusted for the national situation]

How often do you your grocery shopping online? [not reported upon in the current document]
[7-point scale ranging from never to every time]

What describes how you do your grocery shopping best? [not reported upon in the current document]
1. I buy for multiple days at once
2. I buy as many products as possible at once and buy a few products at other times
3. I buy a few products each time

How often do you go grocery shopping? [not reported upon in the current document]
4. 1x per month
5. 2x per month
6. 1x per week
7. 2/3 x per week
8. 4/5x per week
9. Daily

On average, how long does it take you to prepare the main meal of the day (in minutes)? [not reported upon in the current document]
[Answer in minutes]

Finally, we would like to ask you a few questions about the considerations you make. Each time, you will see two different statements. Please indicate which of the two statements is most decisive to you. [items in randomized order] [not reported upon in the current document]
1. The food is healthy
2. The food is tasty

1. The food is healthy
2. The food is easy to prepare

10. The food is healthy
11. I have enough food at home

1. The food is healthy
2. The food is cheap

1. The food is healthy
2. I don't have too much food at home

1. The food is tasty
2. The food is easy to prepare

1. The food is tasty
2. I have enough food at home

1. The food is tasty
2. The food is cheap

1. The food is tasty
2. I don't have too much food at home

1. The food is easy to prepare
2. I have enough food at home

1. The food is easy to prepare
2. The food is cheap

1. The food is easy to prepare
2. I don’t have too much food at home

1. I have enough food at home
2. The food is cheap

1. I have enough food at home
2. I don’t have too much food at home

1. The food is cheap
2. I don’t have too much food at home

Thank you for completing this survey. You have been selected to participate in the full research.
For the second part of this research we would like to ask you to pay close attention to the food and drink products you throw away in the upcoming week: from this Monday [DATE] up to and including Sunday [DATE].

After this week, you will receive a survey with questions about what you have thrown away.

This question will be about: All edible food and drink products you have bought in the (online) (super) market or have home-grown that are thrown away. This also includes products that are spoiled or past their expiration date. It does not matter if you have thrown the food away in the general trashcan, food waste container, compost heap or gave it to an animal (pet, birds, et cetera), or otherwise. It is all included.

It will not be about:
1. Bones, peels, seeds or stumps.
2. Food and drink products that are thrown away when eating in a restaurant or canteen.

Thank you very much in advance!

**Introduction to the second survey**
Last week you received an email to pay close attention to the food and drink product you have thrown away. This questionnaire will be about those products. As a reminder:

This question **will** be about:

- All edible food and drink products you have bought in the (online) (super) market or have home-grown that are thrown away.
- This also includes products that are spoiled or past their expiration date.
- It does not matter if you have thrown the food away in the general trashcan, food waste container, compost heap or gave it to an animal (pet, birds, et cetera), or otherwise. It is all included.

It will **not** be about:
1. Bones, peels, seeds or stumps.
2. Food and drink products that are thrown away when eating in a restaurant or canteen.

**Questionnaire B – food waste measure**

**Food categories**

Question 1: Please tick the boxes of the products that are disposed of in your household in the past week. In case of complete meals, please report the main ingredients separately.

- Fresh vegetables and salads
- Non-fresh vegetables (jar / canned / frozen)
- Fresh fruit
- Non-fresh fruit (jar / canned / dried / frozen)
- Potatoes
- Potato products (fries, chips, baby or precooked potatoes, et cetera)
- Pasta
- Rice and remaining grains (including wraps, couscous, et cetera)
- Beans, lentils, chickpeas, et cetera.
- Meat (please report cold meat slices at “bread toppings”)
- Meat substitute
- Fish
- Bread toppings (cold meats slices, cheese slices, sweet topping, et cetera)
- Bread
- Cereals (muesli, granola, oat, brinta, et cetera)
- Yoghurt, custard, et cetera
- Cheese (cheese cubes, French cheese, sprinkle cheese. Excluded: cheese as bread topping)
- Eggs
- Soups / curry
- Sauce (ketchup, mayonnaise, cocktail sauce, et cetera)
- Candy / cookies / granola bars / chocolate bars
- Crisps / nuts
- Non-alcoholic beverages (milk, juice, soda. Excluded: water, tea, coffee, diluted syrup)
- Alcoholic beverages
- I have not thrown away any food or drink products

**Instructions to the second survey**

Food waste states

We split food waste into several categories, which are explained below. Please read this carefully as these categories will be used in the next questions.

Food waste can be categorised into:

1) Completely unused foods: food that is disposed of which is not used at all. For instance, unopened packages, including unopened parts of multipacks, moulted apples, dried leek, complete bread.

2) Partly used foods: food that is disposed of after it has been party used. For instance, a few bread slices, halve a package of meat cuts, halve an onion or halve a package of milk.

3) Meal leftovers: leftovers that are disposed of after these were left on the plate, pots or pans. For instance, potato mash or rice that is left on the plate or in the pan, sandwiches that were not eaten during lunch.

4) Leftovers after storing: meal leftovers that are disposed of after these were stored in the fridge or freezer to be eaten at a later moment. For instance, a frozen pasta portion of last week.

You will receive several questions about different type of food and drink products you have disposed of in the past week. First, we ask how much of a certain product your household disposed of in the past week. Next, we ask to which category (unused, partly used, meal leftovers, leftover after it was stored) the majority of the disposed of food product belonged when it was disposed of. Please pay attention to which food product if refers!

**Respondents received questions corresponding to their ticked boxes**
Question 2: Fresh vegetables and salads.
In your household, how much fresh vegetables were disposed of in the past week?
*One serving spoon equals 50 gram. As a reference: this is equal to halve a leek or four mushrooms.*

- Less than one serving spoon
- 1 to 2 serving spoons
- 3 to 4 serving spoons
- 5 to 6 serving spoons
- More than 6 serving spoons

Question 3: To which category did the (majority) of the disposed of fresh vegetables and salads belong?
*Please tick the category that occurred the most. You can tick more than one box if multiple categories occurred in the same amount.*

- Completely unused foods: food that is disposed of which is not used at all (e.g., a leek)
- Partly used foods: food that is disposed of after it is party used (e.g., half an union)
- Meal leftovers: meal leftovers that are disposed of after these were left on the plate, pots or pans
- Leftovers after storing: Meal leftovers that are disposed of after these were stored

Question 4: Non-fresh vegetables (jar / canned / frozen).
In your household, how many non-fresh vegetables (jar / canned / frozen) were disposed of in the past week?
*One serving spoon equals 50 gram. As a reference: this is equal to halve a leak or four mushrooms.*

- Less than one serving spoon
- 1 to 2 serving spoons
- 3 to 4 serving spoons
- 5 to 6 serving spoons
- More than 6 serving spoons

Question 5: To which category did the (majority of) disposed of non-fresh vegetables belong?
*Please tick the category that occurred the most. You can tick more than one box if multiple categories occurred in the same amount.*

- Completely unused foods: food that is disposed of which is not used at all (e.g., unopened frozen / canned spinach package)
- Partly used foods: food that is disposed of after it is party used (e.g., half used frozen / canned spinach package)
- Meal leftovers: meal leftovers that are disposed of after these were left on the plate, pots or pans
- Leftovers after storing: Meal leftovers that are disposed of after these were stored

Question 6: Fresh fruit.
In your household, how many fresh fruits were disposed of in the past week?
*One apple or banana is one piece of fruit. In case of small fruits, such as strawberries or grapes, one small bowl is considered 'one piece'.*

- Approximately one fourth of a piece of fruit or less
- Approximately halve a piece of fruit
- Approximately 1 piece of fruit
- 2 to 4 pieces of fruit
- More than 4 pieces of fruit

Question 7: To which category did the (majority of) disposed of fresh fruit belong?
*Please tick the category that occurred the most. You can tick more than one box if multiple categories occurred in the same amount.*

- Completely unused foods: food that is disposed of which is not used at all (e.g., an apple)
- Partly used foods: food that is disposed of after it is party used (e.g., half an apple that is not used in a dish)
- Meal leftovers: meal leftovers that are disposed of after these were left on the plate, pots or pans (e.g., half eaten apple or a fruit salad)
- Leftovers after storing: Meal leftovers that are disposed of after these were stored (e.g. fruit salad after it was stored)

Question 8: Non-fresh fruit (jar / canned / dried / frozen).
In your household, how many non-fresh fruits (jar / canned / dried / frozen) were disposed of in the past week?
*One pear or peach from a can is one piece of fruit. In case of small fruits, as blueberries or tangerine wedges, one small bowl is considered 'one piece'.*

- Approximately one fourth of a piece of fruit or less
- Approximately halve a piece of fruit
- Approximately 1 piece of fruit
- 2 to 4 pieces of fruit
- More than 4 pieces of fruit

Question 9: To which category did the (majority of) disposed of non-fresh fruit belong?
*Please tick the category that occurred the most. You can tick more than one box if multiple categories occurred in the same amount*.

- Completely unused foods: food that is disposed of which is not used at all (e.g., unopened fruit can)
- Partly used foods: food that is disposed of after it is party used (e.g., half full fruit can)
- Meal leftovers: meal leftovers that are disposed of after these were left on the plate, pots or pans (e.g. bowl with fruit)
- Leftovers after storing: Meal leftovers that are disposed of after these were stored (e.g. fruit salad after it was stored)

Question 10: Potatoes
In your household, how many potatoes were disposed of in the past week?
*One serving spoon equals 50 gram. As a reference: this is equal to halve a midsize potato.*

- Less than one serving spoon
- 1 to 2 serving spoons
- 3 to 4 serving spoons
- 5 to 6 serving spoons
- More than 6 serving spoons

Question 11: To which category did the (majority of) disposed of potatoes belong?
*Please tick the category that occurred the most. You can tick more than one box if multiple categories occurred in the same amount.*

- Completely unused foods: food that is disposed of which is not used at all (e.g., complete potato package)
- Partly used foods: food that is disposed of after it is party used (e.g., half a potato package)
- Meal leftovers: meal leftovers that are disposed of after these were left on the plate, pots or pans (e.g. smashed potato)
- Leftovers after storing: meal leftovers that are disposed of after these were stored (e.g. smashed potato after it was stored)

Question 12: Potato products (fries, baby potatoes, precooked potatoes, et cetera).
In your household, how many potato products (fries, precooked potatoes, et cetera) were disposed of in the past week?

- Less than 10 fries / baby potatoes / pieces
- 10 to 25 fries / baby potatoes / pieces
- More than 25 fries / baby potatoes / pieces (approximately half a package of 500 gram)
- Full package (750 gram) fries / baby potatoes / pieces
- More than a package (750 gram) fries / baby potatoes / pieces

Question 13: To which category did the (majority of) disposed of potato products belong?
*Please tick the category that occurred the most. You can tick more than one box if multiple categories occurred in the same amount.*

- Completely unused foods: food that is disposed of which is not used at all (e.g., complete potato fries package)
- Partly used foods: food that is disposed of after it is party used (e.g., half a potato fries package)
- Meal leftovers: meal leftovers that are disposed of after these were left on the plate, pots or pans
- Leftovers after storing: meal leftovers that are disposed of after these were stored

Question 14: Pasta
In your household, how much pasta was disposed of in the past week?
*One serving spoon equals 50 gram.*

- Less than one serving spoon
- 1 to 2 serving spoons
- 3 to 4 serving spoons
- 5 to 6 serving spoons
- More than 6 serving spoons

Question 15: To which category did the (majority of) disposed of pasta belong?
*Please tick the category that occurred the most. You can tick more than one box if multiple categories occurred in the same amount.*

- Completely unused foods: food that is disposed of which is not used at all (e.g., complete pasta package)
- Partly used foods: food that is disposed of after it is party used (e.g., half pasta package)
- Meal leftovers: meal leftovers that are disposed of after these were left on the plate, pots or pans
- Leftovers after storing: meal leftovers that are disposed of after these were stored

Question 16: Rice and remaining grains (including wraps, couscous, et cetera).
In your household, how much rice and remaining grains (including wraps, couscous, et cetera) was disposed of in the past week?
*One serving spoon equals 50 gram.*

- Less than one serving spoon
- 1 to 2 serving spoons
- 3 to 4 serving spoons
- 5 to 6 serving spoons
- More than 6 serving spoons

Question 17: To which category did the (majority of) disposed of rice belong?
*Please tick the category that occurred the most. You can tick more than one box if multiple categories occurred in the same amount.*

- Completely unused foods: food that is disposed of which is not used at all (e.g., complete rice package)
- Partly used foods: food that is disposed of after it is party used (e.g., half rice package)
- Meal leftovers: meal leftovers that are disposed of after it was left on the plate, pots or pans
- Leftovers after storing: meal leftovers that are disposed of after it was stored

Question 18: Beans, lentils, chickpeas, et cetera.
In your household, how much beans, lentils, chickpeas, et cetera were disposed of in the past week?

- Less than one serving spoon
- 1 to 2 serving spoons
- 3 to 4 serving spoons
- 5 to 6 serving spoons
- More than 6 serving spoons

Question 19: To which category did the (majority of) disposed of beans, lentils, chickpeas, et cetera belong?
*Please tick the category that occurred the most. You can tick more than one box if multiple categories occurred in the same amount.*

- Completely unused foods: food that is disposed of which is not used at all (e.g., unopened bean jar)
- Partly used foods: food that is disposed of after it is party used (e.g., half full bean jar)
- Meal leftovers: meal leftovers that are disposed of after it was left on the plate, pots or pans
- Leftovers after storing: meal leftovers that are disposed of after it was stored

Question 20: Meat.
In your household, how much meat was disposed of in the past week?
*A portion refers to one chicken breast, one steak, et cetera. In case of smaller pieces, as minced meat, try to estimate it in whole pieces of meat (e.g., one package of minced meat is equal to two portions).*

- Approximately half a portion or less
- Approximately one portion
- 2 to 3 portions
- 4 to 5 portions
- More than 5 portions

Question 21: To which category did the (majority of) disposed of meat belong?
*Please tick the category that occurred the most. You can tick more than one box if multiple categories occurred in the same amount.*

- Completely unused foods: food that is disposed of which is not used at all (e.g., sausage package)
- Partly used foods: food that is disposed of after it is party used (e.g., half a sausage package)
- Meal leftovers: meal leftovers that are disposed of after these were left on the plate, pots or pans
- Leftovers after storing: meal leftovers that are disposed of after these were stored

Question 22: Meat substitutes.
In your household, how much meat substitutes were disposed of in the past week?
*A portion refers to a vegetarian burger, et cetera. In case of smaller pieces, as minced meat, try to estimate it in whole pieces of meat (e.g., one package of minced vegetarian meat is equal to two portions).*

- Approximately half a portion or less
- Approximately one portion
- 2 to 3 portions
- 4 to 5 portions
- More than 5 portions

Question 23: To which category did the (majority of) disposed of meat substitutes belong?
*Please tick the category that occurred the most. You can tick more than one box if multiple categories occurred in the same amount.*

- Completely unused foods: food that is disposed of which is not used at all (e.g., vegetarian burger package)
- Partly used foods: food that is disposed of after it is party used (e.g., half a vegetarian burger package)
- Meal leftovers: meal leftovers that are disposed of after these were left on the plate, pots or pans
- Leftovers after storing: meal leftovers that are disposed of after these were stored

Question 24: Fish.
In your household, how much fish was disposed of in the past week?
*A portion refers to one fish filled, one piece of salmon, et cetera.*

- Approximately half a portion or less
- Approximately a complete portion
- 2 to 3 portions
- 4 to 5 portions
- More than 5 portions

Question 25: To which category did the (majority of) disposed of fish belong?
*Please tick the category that occurred the most. You can tick more than one box if multiple categories occurred in the same amount.*

- Completely unused foods: food that is disposed of which is not used at all (e.g., complete fish package)
- Partly used foods: food that is disposed of after it is party used (e.g., half a fish package)
- Meal leftovers: meal leftovers that are disposed of after these were left on the plate, pots or pans
- Leftovers after storing: meal leftovers that are disposed of after these were stored

Question 26: Bread toppings (cold meats slices, cheese slices, sweet topping, et cetera).
In your household, how much bread toppings (cold meats slices, cheese slices, sweet topping, et cetera) were disposed of in the past week?
*One portion is what is used on one slice of bread / sandwich / portion of baguette.*

- Approximately half a portion or less
- Approximately a complete portion
- 2 to 3 portions
- 4 to 5 portions
- More than 5 portions

Question 27: To which category did the (majority of) disposed of bread toppings belong?
*Please tick the category that occurred the most. You can tick more than one box if multiple categories occurred in the same amount.*

- Completely unused foods: food that is disposed of which is not used at all (e.g., complete package with meat slices)
- Partly used foods: food that is disposed of after it is party used (e.g., half a package with meat slices)
- Meal leftovers: meal leftovers that are disposed of after these were left on the plate, pots or pans
- Leftovers after storing: meal leftovers that are disposed of after these were stored

Question 28: Bread.
In your household, how much bread was disposed of in the past week?
*A (raisin) bun, portion of baguette or sandwich is similar to one slice of bread.*

- Less than one slice of bread
- One or a few slices of bread
- Approximately half a loaf
- Approximately one loaf
- More than one loaf

Question 29: To which category did the (majority of) disposed of bread belong?
*Please tick the category that occurred the most. You can tick more than one box if multiple categories occurred in the same amount*.

- Completely unused foods: food that is disposed of which is not used at all (e.g., whole loaf)
- Partly used foods: food that is disposed of after it is party used (e.g., slices of bread)
- Meal leftovers: meal leftovers that are disposed of after these were left on the plate, pots or pans (e.g., bread crusts)
- Leftovers after storing: meal leftovers that are disposed of after these were stored

Question 30: Cereal (muesli, granola, oat, porridge, et cetera).
In your household, how much cereal (muesli, granola, oat, porridge, et cetera) was disposed of in the past week?
*A portion is the amount of cereals used for one bowl of breakfast.*

- Less than half a portion
- A half to one and a half portion
- Multiple portions (approximately half a package)
- Approximately a complete package
- Multiple packages

Question 31: To which category did the (majority of) disposed of cereals belong?
*Please tick the category that occurred the most. You can tick more than one box if multiple categories occurred in the same amount.*

- Completely unused foods: food that is disposed of which is not used at all (e.g., complete cereal package)
- Partly used foods: food that is disposed of after it is party used (e.g., half a cereal package)
- Meal leftovers: meal leftovers that are disposed of after these were left on the plate, pots or pans
- Leftovers after storing: meal leftovers that are disposed of after these were stored

Question 32: Yoghurt, custard, et cetera.
In your household, how much yoghurt, custard, et cetera was disposed of in the past week?
*A portion is a small bowl with yoghurt / custard / et cetera.*

- Less than half a portion
- A half to one and a half portion
- Multiple portions (approximately half a litre package)
- Approximately a complete litre package
- Multiple litre packages

Question 33: To which category did the (majority of) disposed of yoghurt, custard, et cetera belong?
*Please tick the category that occurred the most. You can tick more than one box if multiple categories occurred in the same amount.*

- Completely unused foods: food that is disposed of which is not used at all (e.g., complete yoghurt package)
- Partly used foods: food that is disposed of after it is party used (e.g., half a yoghurt package)
- Meal leftovers: meal leftovers that are disposed of after these were left on the plate, pots or pans
- Leftovers after storing: meal leftovers that are disposed of after these were stored

Question 34: Cheese (cheese dices, French cheese, sprinkle cheese; excluded: cheese as bread topping).
In your household, how much cheese (cheese dices, French cheese, sprinkle cheese; excluded: cheese as bread topping) was disposed of in the past week?
*A handful of cheese can be seen as a dice of cheese.*

- Less than one dice of cheese
- Approximately one dice of cheese
- 1 to 3 cheese dices
- 4 to 5 cheese dices
- More than 5 cheese dices

Question 35: To which category did the (majority of) disposed of cheese belong?
*Please tick the category that occurred the most.*
*You can tick more than one box if multiple categories occurred in the same amount.*

- Completely unused foods: food that is disposed of which is not used at all (e.g., complete French cheese)
- Partly used foods: food that is disposed of after it is party used (e.g., partly used French cheese)
- Meal leftovers: meal leftovers that are disposed of after these were left on the plate, pots or pans
- Leftovers after storing: meal leftovers that are disposed of after these were stored

Question 36: Eggs.
In your household, how many eggs were disposed of in the past week?

- Less than 1 egg
- 1 egg
- 2 to 3 eggs
- 4 to 5 eggs
- More than 5 eggs

Question 37: To which category did the (majority of) disposed of eggs belong?
*Please tick the category that occurred the most. You can tick more than one box if multiple categories occurred in the same amount.*

- Completely unused foods: food that is disposed of which is not used at all (e.g., complete eggs)
- Partly used foods: food that is disposed of after it is party used (e.g., egg white)
- Meal leftovers: meal leftovers that are disposed of after these were left on the plate, pots or pans
- Leftovers after storing: meal leftovers that are disposed of after these were stored

Question 38: Soups / curry.
In the household, how much soup / curry was disposed of in the past week?

- Less than half a ladle
- Half to one and a half ladle
- Multiple ladles (approximately half a litre)
- Approximately 1 litre
- More than 1 litre

Question 39: To which category did the (majority of) disposed of soup belong?
*Please tick the category that occurred the most. You can tick more than one box if multiple categories occurred in the same amount.*

- Completely unused foods: food that is disposed of which is not used at all (e.g., complete soup package). Not applicable in case of home-made soup
- Partly used foods: food that is disposed of after it is party used (e.g., half a soup package). Not applicable in case of home-made soup
- Meal leftovers: meal leftovers that are disposed of after these were left on the plate, pots or pans (warmed package of soup or home-made soup)
- Leftovers after storing: meal leftovers that are disposed of after these were stored

Question 40: Sauces (ketchup, mayonnaise, cocktail sauce, et cetera).
In your household, how much sauce (ketchup, mayonnaise, cocktail sauce, et cetera) was disposed of in the past week?
*One tablespoon equals 15 grams.*

- Less than a table spoon
- 1 to 3 table spoons
- Multiple table spoons
- Approximately half a jar / bottle
- More than one jar / bottle

Question 41: To which category did the (majority of) disposed of sauces belong?
*Please tick the category that occurred the most. You can tick more than one box if multiple categories occurred in the same amount*.

- Completely unused foods: food that is disposed of which is not used at all (e.g., complete sauce jar)
- Partly used foods: food that is disposed of after it is party used (e.g., half a sauce jar)
- Meal leftovers: meal leftovers that are disposed of after these were left on the plate, pots or pans
- Leftovers after storing: meal leftovers that are disposed of after these were stored

Question 42: Candy / cookies / granola bars / chocolate bars.
In your household, how much candy / cookies / granola bars / chocolate bars were disposed of in the past week?
*A portion is a handful of sweets, small chocolate bar, a cookie, et cetera.*

- Approximately half a portion or less
- Approximately one portion
- 2 to 3 portions
- 4 to 5 portions
- More than 5 portions

Question 43: To which category did the (majority of) disposed of candy belong?
*Please tick the category that occurred the most. You can tick more than one box if multiple categories occurred in the same amount.*

- Completely unused foods: food that is disposed of which is not used at all (e.g., one cookie package)
- Partly used foods: food that is disposed of after it is party used (e.g., half a cookie package)
- Meal leftovers: meal leftovers that are disposed of after these were left on the plate, pots or pans
- Leftovers after storing: meal leftovers that are disposed of after these were stored

Question 44: Crisps / nuts.
In your household, how much crisps / nuts were disposed of in the past week?
*A portion is a handful of crisps or nuts.*

- Approximately half a portion or less
- Approximately one portion
- 2 to 3 portions
- 4 to 5 portions
- More than 5 portions

Question 45: To which category did the (majority of) disposed of crisps / nuts belong?
*Please tick the category that occurred the most. You can tick more than one box if multiple categories occurred in the same amount.*

- Completely unused foods: food that is disposed of which is not used at all (e.g., bag of crisps)
- Partly used foods: food that is disposed of after it is party used (e.g., half a bag of crisps)
- Meal leftovers: meal leftovers that are disposed of after these were left on the plate, pots or pans
- Leftovers after storing: meal leftovers that are disposed of after these were stored

Question 46: Non-alcoholic beverages (milk, juice, soda; excluded: water, tea, coffee, diluted syrup).
In your household, how much non-alcoholic beverages (milk, juice, soda; excluded: water, tea, coffee, diluted syrup) was disposed of in the past week?

- Less than half a glass
- A half to one and a half glass
- Multiple glasses (approximately half a litre)
- Approximately one litre
- More than one litre

Question 47: To which category did the (majority of) non-alcoholic beverages belong?
*Please tick the category that occurred the most. You can tick more than one box if multiple categories occurred in the same amount.*

- Completely unused foods: drinks that are disposed of which are not used at all (e.g., a milk package)
- Partly used foods: drinks that is disposed of after it is party used (e.g., half a milk package)
- Meal leftovers: beverage that is left in the glass
- Leftovers after storing: meal leftovers that are disposed of after these were stored

Question 48: Alcoholic beverages.
In your household, how many alcoholic beverages were disposed of in the past week?

- Less than half a beer glass
- Half to one and a half beer glass
- Multiple beer glasses (approximately half a litre)
- Approximately one litre
- More than one litre

Question 49: To which category did the (majority of) alcoholic beverages belong?
*Please tick the category that occurred the most. You can tick more than one box if multiple categories occurred in the same amount.*

Completely unused foods: drinks that are disposed of which are not used at all (e.g., a bottle of wine)
Partly used foods: drinks that are disposed of after it is party used (e.g., half a bottle of wine)
Meal leftovers: beverage that is left in the glass
Leftovers after storing: beverage leftovers that are disposed of after these were stored

Thank you very much for completing this questionnaire. In case you of any comments, you can type these in the space below.

**NOTE**: The food examples given in each question were adjusted where needed to match the country specific situation and enlarge the respondents' understanding.

**Supplementary material (S2) – Table S1:**
**Conversion table for calculation of the food waste measure, in gram developed by van Geffen et al. 2017 (23)**

This list indicates for each answer option provided for the questions in the second survey (related to how much was wasted in the categories), the number of grams that was used in the calculations.

1) Vegetable = 25.
2) Vegetable = 75.
3) Vegetable = 150.
4) Vegetable = 250.
5) Vegetable = 350.

1) Non fresh veg = 25.
2) Non fresh veg = 75.
3) Non fresh veg = 150.
4) Non fresh veg = 250.
5) Non fresh veg = 350.

1) Fruit = 25.
2) Fruit = 50.
3) Fruit = 100.
4) Fruit = 300.
5) Fruit = 500.

1) Non-fresh fruit = 20.
2) Non-fresh fruit = 40.
3) Non-fresh fruit = 80.
4) Non-fresh fruit = 240.
5) Non-fresh fruit = 400.

1) Potato = 25.
2) Potato = 75.
3) Potato = 150.
4) Potato = 250.
5) Potato = 350.

1) Potato Prod = 25.
2) Potato Prod = 88.
3) Potato Prod = 375.
4) Potato Prod = 750.
5) Potato Prod = 1125.

1) Pasta = 25.
2) Pasta = 75.
3) Pasta = 150.
4) Pasta = 250.
5) Pasta = 350.

1) Rice = 25.
2) Rice = 75.
3) Rice = 150.
4) Rice = 250.
5) Rice = 350.

1) Legumes = 25.
2) Legumes = 75.
3) Legumes = 150.
4) Legumes = 250.
5) Legumes = 350.

1) Meat = 75.
2) Meat = 150.
3) Meat = 375.
4) Meat = 675.
5) Meat = 900.

1) Meat sub = 45.
2) Meat sub = 90.
3) Meat sub = 225.
4) Meat sub = 405.
5) Meat sub = 540.

1) Fish = 75.
2) Fish = 150.
3) Fish = 375.
4) Fish = 675.
5) Fish = 900.

1) Topping = 10.
2) Topping = 20.
3) Topping = 50.
4) Topping = 90.
5) Topping = 120.

1) Bread = 18.
2) Bread = 35.
3) Bread = 400.
4) Bread = 800.

5) Bread = 1200.

1) Cereal = 10.
2) Cereal = 40.
3) Cereal = 250.
4) Cereal = 500.
5) Cereal = 1000.

1) Yoghurt = 38.
2) Yoghurt = 150.
3) Yoghurt = 500.
4) Yoghurt = 1000.
5) Yoghurt = 2000.

1) Cheese = 5.
2) Cheese = 10.
3) Cheese = 20.
4) Cheese = 45.
5) Cheese = 60.

1) Egg = 30.
2) Egg = 60.
3) Egg = 150.
4) Egg = 270.
5) Egg = 360.

1) Soup = 38.
2) Soup = 150.
3) Soup = 500.
4) Soup = 1000.
5) Soup = 1500.

1) Sauce = 10.
2) Sauce = 30.
3) Sauce = 90.
4) Sauce = 225.
5) Sauce = 675.

1) Candy = 10.
2) Candy = 20.
3) Candy = 50.
4) Candy = 90.
5) Candy = 120.

1) Chips = 10.
2) Chips = 20.
3) Chips = 50.
4) Chips = 90.
5) Chips = 120.

1) Non-alcohol = 68.
2) Non-alcohol = 250.
3) Non-alcohol = 500.

4) Non-alcohol = 1000.
5) Non-alcohol = 1500.

1) Alcohol = 75.
2) Alcohol = 300.
3) Alcohol = 500.
4) Alcohol = 1000.
5) Alcohol = 1500.
